# Flexoelectricity and the fluctuations of (active) living cells: Implications for energy harvesting, ion transport, and neuronal activity

**DOI:** 10.1093/pnasnexus/pgaf362

**Published:** 2025-12-16

**Authors:** Pratik Khandagale, Liping Liu, Pradeep Sharma

**Affiliations:** Department of Mechanical and Aerospace Engineering, University of Houston, 4226 Martin Luther King Boulevard, Houston, TX 77204, USA; Departments of Mathematics, and Department of Mechanical and Aerospace Engineering, Rutgers University, Piscataway, NJ 08854, USA; Departments of Physics, Mechanical and Aerospace Engineering, and the Materials Science and Engineering Program, University of Houston, 4226 Martin Luther King Boulevard, Houston, TX 77204, USA

**Keywords:** living biological cells, flexoelectricity, nonequilibrium statistical mechanics, active ion transport and energy harvesting, neuronal activation function

## Abstract

Biological membranes universally exhibit flexoelectricity, a form of electromechanical coupling in which membrane curvature induces electric polarization. This phenomenon enables the conversion of mechanical deformations into electrical signals and plays a central role in sensory processes such as hearing. Flexoelectricity can also ostensibly provide a facile route for energy harvesting via membrane flexure, and, in principle, enable useful work (e.g. as an ionic pump). While all cell membranes undergo noticeable thermal fluctuations at physiological temperatures, equilibrium fluctuations alone cannot yield net harvested energy. In this work, we recognize that cells are inherently active, living systems, driven far from equilibrium by processes such as protein dynamics and ATP hydrolysis, and develop a theoretical framework to investigate the flexoelectric response of actively fluctuating membranes. Our results reveal that activity can significantly amplify transmembrane voltage and polarization, suggesting a physical mechanism for energy harvesting and directed ion transport in living cells. We highlight potential applications of our findings in the context of ion transport and neuronal action potentials.

Significance Statement Living cells constantly experience nanoscale membrane fluctuations due to molecular motion and activity. Can these fluctuations produce electricity? At first glance, the answer appears to be no: classical thermodynamics prohibits net energy extraction from equilibrium thermal noise. However, cells are not passive systems—they are driven by internal active processes such as protein activity and ATP consumption. We show that these active fluctuations, when coupled with the universal electromechanical property of flexoelectricity, can generate transmembrane voltages and even drive ion transport. Our theoretical framework reveals how living membranes may harvest mechanical energy to perform electrical work, offering a new lens to understand sensory processes, neuronal firing, and the broader interface between mechanics and bioelectricity in life.

## Introduction

In a biological cell, mechanical deformation is often intimately coupled to electrical fields. Indeed, this interplay makes its presence felt in a variety of physiological processes, including cellular communication (1, 2), endocytosis (3, 4), the electromechanical transduction underlying the hearing mechanism (5–8), cell fusion (9) and electroporation (10 , 11), proprioception (12 ), osmoregulation (13), among others. We refer the reader to several review and expository articles on the confluence of mechanics and electromagnetic fields in biological cells (14–17 ).

**Fig. 1.**
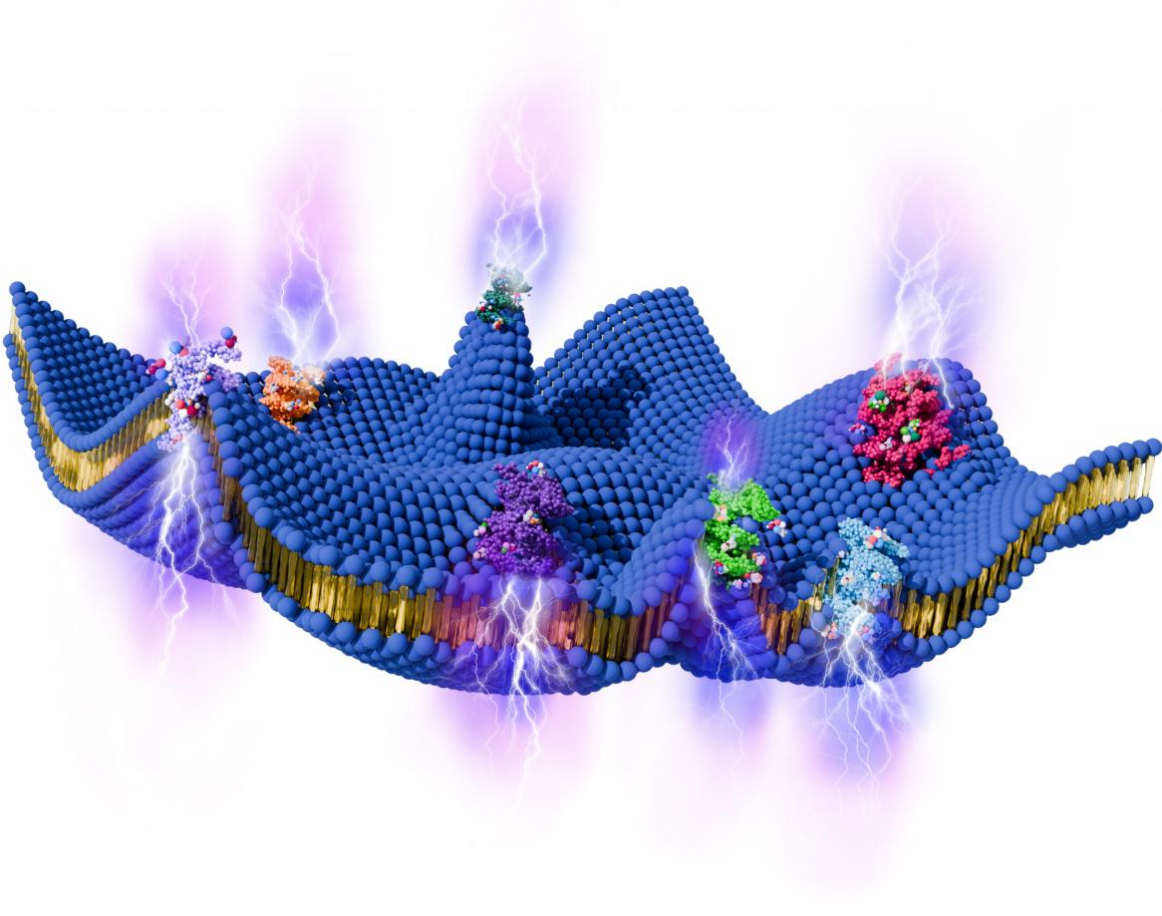
Schematic of an active cell membrane. In a typical active biological process, active proteins (shown in a variety of colors) in a cell membrane interact with various biological components, such as the ATP molecules. These interactions of active proteins generate active noise (fluctuation) force within a cell membrane, mechanically affecting the out-of-plane displacement of a cell membrane. Due to the flexoelectric coupling of a cell membrane, changes in out-of-plane displacement induce changes in the transmembrane voltage of a cell membrane, resulting in energy harvesting, active transport of ions, and the generation of electric current across the cell membrane.

Piezoelectricity is the most extensively studied form of electromechanical coupling in the physical sciences, yet it may be the least relevant at the cellular scale. Its manifestation requires a lack of microstructural symmetry—a condition rarely met in soft, fluidic membranes. By contrast, electrostriction (or the Maxwell stress effect) is universal: even modest electric fields can induce measurable deformations in soft cellular membranes. However, this effect represents a one-way coupling—an electric field can generate deformation, but not the reverse. Moreover, since the induced deformation scales with the square of the electric field, it is insensitive to polarity. Most notably, the potential for “energy harvesting,” i.e. the conversion of mechanical energy into electricity, is minimal.

It is therefore unsurprising that flexoelectricity has recently emerged as a particularly significant form of electromechanical coupling in biological systems. Flexoelectricity is universally present in all dielectrics and describes a direct, bidirectional coupling between strain gradients and polarization. Given that biological membranes are highly susceptible to bending, flexoelectricity offers a direct and linear bridge between mechanical forces and electrical cues.

Pioneering work by Petrov and collaborators has proposed flexoelectricity as a fundamental mechanism underlying electromechanical coupling in living cells (18–20), with implications for hearing (21), ion transport (22), and neuronal activity (23). Subsequent studies by Brownell, Raphael, and others—including one of the authors of this work—have demonstrated that flexoelectricity plays a key role in the electromotility of hair bundles (24, 25), a process essential not only for hearing but also for musical perception (8).

Building on the preceding discussion of electromechanical coupling, we note that a foundational feature of membrane physics is the presence of shape fluctuations. At physiological temperatures, lipid bilayers exhibit continuous thermal undulations, giving rise to a rich and dynamic spectrum of curvature variations (26–32). Though passive in origin, these fluctuations couple with membrane proteins, cause directed motion (33), modulate critical cellular processes such as fusion and budding, and generate measurable voltage signals through flexoelectric effects (34, 35).

The statistical mechanics governing these thermal fluctuations is well established and has been captured in theoretical models that relate bending rigidity, surface tension, and dielectric properties to the spectral characteristics of membrane deformation and polarization (36–47).

Equilibrium thermal fluctuations are not correlated in time and are governed by the fluctuation—dissipation theorem (39). With just thermal fluctuations, the governing Langevin-type dynamics (see 5) of the cell membrane satisfy time reversibility and detailed balance (39, 48, 49). Due to this, equilibrium thermal fluctuations cannot drive directed processes or yield net energy transduction. For flexoelectric membranes, this means that while momentary polarization may occur due to curvature, no sustained voltage or current can be extracted from purely thermal noise (fluctuation). This thermodynamic constraint effectively precludes equilibrium membranes from powering active biological functions or operating as viable energy harvesters. In contrast, active noise, e.g. arising from ATP hydrolysis or molecular motor activity, is correlated in time and violates the detailed balance while breaking the time-reversal symmetry of the cell membrane dynamics. Hence, active noise is capable of injecting energy into the system (39, 48, 49) (see Fig. 1).

Despite significant advances, the role of active fluctuations in shaping electromechanical coupling—especially flexoelectric effects—remains insufficiently understood. A central open question is whether noise arising from active molecular processes can drive a sustained and directional electrical response in membranes, and under what specific physical or biochemical conditions such a response becomes energetically consequential. Can living membranes transduce mechanical activity into ionic transport or initiate nonlinear electrical events? How do stochastic fluctuations give rise to measurable bioelectric signatures, and might these signatures be harnessed to perform mechanical work, encode information, or regulate cellular signaling networks?

To explore these questions, we develop a theoretical framework that approximates the otherwise intractable nonequilibrium statistical mechanics problem, enabling the study of actively fluctuating flexoelectric membranes. Building on prior works in elastic membrane theory (26–28), statistical thermodynamics of soft matter (39, 47, 50–54), and flexoelectric modeling (34, 35, 55–57), we incorporate active noise as a temporally correlated stochastic process that drives out-of-plane membrane displacement. Our formulation considers both passive thermal and active fluctuations (58–61), and quantifies their respective contributions to renormalized material constants (e.g. bending modulus, dielectric coefficients, flexoelectric coupling) at steady state. We specifically follow the treatment by Kulkarni (42) (which focused on the purely mechanical problem) and interlace the specific nuances of incorporating flexoelectricity.

By computing the fluctuation-induced corrections to the membrane Hamiltonian and solving the coupled Langevin-type equations for polarization and displacement, we demonstrate that active fluctuations can lead to amplified, sustained transmembrane voltages. These voltages exhibit nonlinear dependencies on curvature and activity strength, resembling the threshold behavior seen in neuronal action potentials and artificial neural network activations (62–64). We show that the harvested electrical energy scales with curvature-induced active forces, and derive conditions under which the polarity and magnitude of membrane potential changes are sufficient to trigger biologically relevant transport.

Furthermore, we propose a biophysically consistent mechanism for *active ion transport* through flexoelectric membranes. Depending on the direction of voltage modulation and membrane polarization, the system can perform effective proton pumping, transferring ions against the resting potential gradient using only internal mechanical activity. This result aligns with recent experimental and theoretical work on mechano-electrical transduction and suggests new ways to interpret voltage generation in neurons, cilia, and other excitable structures (42, 47, 65–67).

### Overview of the approach

As is customary in nonequilibrium statistical mechanics, we capture the coupled electromechanical fluctuations driven by active forces and stochastic noise through Langevin equations. Solving these equations yields the joint spectra of our key observables—namely, membrane polarization and out-of-plane displacement. Here, our central approximation comes into play: we must decide how to translate those spectral solutions into concrete measures of energy harvesting or macroscopic membrane response. To do so, we leverage the well-known fact that equilibrium thermal fluctuations allow one to define an effective free energy—and hence compute “renormalized” material constants (bending modulus, dielectric permittivity, flexoelectric coefficient, etc.).

Although a true free energy is undefined out of equilibrium, we adopt it as an instantaneous, energy-like functional whose coefficients incorporate both thermal and active contributions. This surrogate free energy then provides a transparent route to predict how active forces reshape membrane mechanics at steady state. Finally, since our interest lies in mechanical-to-electrical transduction, we simplify by omitting active noise in the polarization channel (retaining only its thermal fluctuations) and focus solely on active, out-of-plane membrane undulations.

## Theoretical formulation

Consider a cell membrane of in-plane size *L* and thickness $t_{m}$. To account for flexoelectricity, the membrane is described by state variables $\left(P_{x},P_{y},P_{z},h\right)$, where $P_{x}$, $P_{y},P_{z}$ are *x*, *y*, *z* components of the polarization (per unit area), respectively, and *h* is the out-of-plane displacement of the mid-plane of the cell membrane. Suppose that at the zero temperature, the membrane occupies the domain $S\;:\;=(0,L{)}^{2}$ on the *xy*-plane. Denote by (34)


(1) $$\begin{array}{cc} & J_{h}=\sqrt{1+|\nabla h{|}^{2}},\;n_{h}=\frac{\left(-\nabla h,1\right)}{J_{h}},\\ & K_{h}=\nabla \cdot \left[\frac{\nabla h}{J_{h}}\right],\;G_{h}=\frac{\text{det}(\nabla \nabla h)}{J_{h}^{4}}, \end{array}$$

where $J_{h}$ is the Jacobian measuring the area of the deformed membrane relative to the flat reference *S*, $n_{h}$ is the unit normal vector on the membrane, and $K_{h}(G_{h})$ is the total (Gaussian) curvature, respectively. The transmembrane resting electric field is denoted by $E_{z}^{0}=E_{z}^{0}\hat{z}$ and is assumed to be constant, where $\hat{z}$ is a unit orientation vector pointing in +ve *z* direction. We propose the following Hamiltonian to describe the elastic-flexoelectric cell membrane (34):


(2) $$\begin{array}{rl} & H[P,h]=\int_{S}[\frac{a}{2}\left(P_{x}^{2}+P_{y}^{2}\right)+\frac{a_{z}}{2}P_{z}^{2}-E_{z}^{0}P_{z}\\ & \;+fP\cdot n_{h}K_{h}+\frac{1}{2}\kappa_{b}(K_{h}{)}^{2}+\kappa_{g}G_{h}+\lambda ]J_{h}. \end{array}$$

Here, $P=[P_{x},P_{y},P_{z}]$ is the polarization (per unit area) of the cell membrane. The term $\frac{a}{2}(P_{x}^{2}+P_{y}^{2})+\frac{a_{z}}{2}P_{z}^{2}$ in 2 accounts for the polarization and associated nonlocal electric field energy (34). The term $-E_{z}^{0}P_{z}$ corresponds to the electrical energy of the cell membrane due to induced polarization $P_{z}$ and resting transmembrane electric field $E_{z}^{0}=E_{z}^{0}\hat{z}$. Assuming the cell membrane is a linear dielectric with permittivity ϵ, and the polarization P varies slowly on S (i.e. the long wavelength limit), the electric constants of the cell membrane are given by Ref. (34)


(3) $$a=\frac{1}{\left(\epsilon -\epsilon_{0}\right)t_{m}},\;a_{z}=\frac{1}{(\epsilon -\epsilon_{0})t_{m}}+\frac{1}{\epsilon_{0}t_{m}}.$$

The term $\frac{1}{2}\kappa_{b}(K_{h}{)}^{2}+\kappa_{g}G_{h}$ in 2 is the Helfrich–Canham bending energy (27, 28, 68, 69), where $\kappa_{b}$ is the bending modulus^a^ and $\kappa_{g}$ is the saddle splay modulus of the cell membrane. The moduli $\kappa_{b}$ and $\kappa_{g}$ represent the energetic costs associated with changes in $K_{h}$ (total curvature) and $G_{h}$ (Gaussian curvature), respectively. The electromechanical coupling term $fP\cdot n_{h}K_{h}$ arises from the flexoelectric effect. Here, the coefficient *f* dictates the strength of the electromechanical coupling. The unit normal vector $n_{h}$ points towards out-of-plane displacement (h) of the cell membrane. Parameter *λ* is surface tension in the cell membrane. We simplify the Hamiltonian in 2 by keeping just the quadratic terms in P and *h* as (34):


(4) $$\begin{array}{rl} & H[P,h]=\lambda L^{2}+\int_{S}[\frac{a}{2}\left(P_{x}^{2}+P_{y}^{2}\right)+\frac{a_{z}}{2}P_{z}^{2}-E_{z}^{0}P_{z}\\ & \;+fP_{z}\Delta h+\frac{1}{2}\kappa_{b}(\Delta h{)}^{2}+\kappa_{g}\text{det}(\nabla \nabla h)+\frac{1}{2}\lambda |\nabla h{|}^{2}]. \end{array}$$

### Time evolution of living flexoelectric cell

We consider the stochastic evolution of state variables of the cell membrane. We consider the polarization ($P_{\mathrm{total}}$) and the out-of-plane displacement $\left(h_{\mathrm{total}}\right)$ of the cell membrane as state variables and decompose them as, $\left(P_{\mathrm{total}},h_{\mathrm{total}})=(\bar{P},\bar{h})+(P,h\right)$, where $\left(\bar{P},\bar{h}\right)$ represent the average values and (P,h) represent the fluctuating part. We introduce the state variable vector composed of the fluctuating part $\left(h,P_{z}\right)$ as:


$$v(r,t)\;:\;=\left[\begin{array}{c} h(r,t)\\ P_{z}(r,t) \end{array}\right],$$

where r=(x,y) is the position vector of the point on the planar cell membrane and *t* denotes time. In account of both the active and thermal noises and in the overdamped regime, the time evolution of v(r,t) should satisfy the Langevin-type equation (42):


(5) $$\begin{array}{rl} \frac{\partial v}{\partial t}(r,t) & =\int_{R^{2}}\Lambda (r-r^{\prime })\left(-\frac{\delta H}{\delta v}(r^{\prime },t\right)\\ & \;+\xi^{\mathrm{th}}(r^{\prime },t)+\xi^{a}(r^{\prime },t))dr^{\prime }. \end{array}$$

Here, $-\frac{\delta H}{\delta v}$ is a conservative force term obtained using 4 as^b^:


(6) $$\begin{array}{rl} -\frac{\delta H}{\delta v} & =-\left[\begin{array}{c} \frac{\delta H}{\delta h}\\ \frac{\delta H}{\delta P_{z}} \end{array}\right]\\ & =-\left[\begin{array}{c} \;f\Delta P_{z}(r,t)+\kappa_{b}\Delta \Delta h(r,t)-\lambda \Delta h(r,t)\\ a_{z}P_{z}(r,t)+f\Delta h(r,t) \end{array}\right]. \end{array}$$

Also, the linear integral operator $(\cdot )\mapsto \int_{R^{2}}\Lambda (r-r^{\prime })(\cdot )dr^{\prime }$ in 5 encodes the dissipative mechanisms and dynamic feedback that depend on the viscosity and electrical conductivity of the membrane and ambient medium. The 2D random processes $\xi^{\mathrm{th}}(r^{\prime },t)$ and $\xi^{a}(r^{\prime },t)$ represent the thermal and active noises, respectively.

Since the kernel $\Lambda \;:\;R^{2}\to R^{2\times 2}$ depends only on the difference $\left(r-r^{\prime }\right)$, applying Fourier transformation with respect to spatial variables r allows us to rewrite 5 for the Fourier modes $v_{q}(t)$ as:


(7) $$\frac{\partial v_{q}(t)}{\partial t}=\Theta_{q}v_{q}(t)+F_{q}^{\mathrm{th}}(t)+F_{q}^{a}(t),$$

where matrix $\Theta_{q}$, thermal noise force vector $F_{q}^{\mathrm{th}}(t)$, and active noise force vector $F_{q}^{a}(t)$ are given by:


(8) $$\begin{array}{cc} & \Theta_{q}=-\Lambda_{q}\left[\begin{array}{cc} \left(\kappa_{b}q^{4}+\lambda q^{2}\right) & -fq^{2}\\ -fq^{2} & a_{z} \end{array}\right],\\ & \left(F_{q}^{\mathrm{th}}(t),F_{q}^{a}(t)\right)=\Lambda_{q}\left(\xi_{q}^{\mathrm{th}}(t),\xi_{q}^{a}(t)\right). \end{array}$$

Here, we define q:=|q|.

A few remarks are in order regarding the dissipation model—specifically, the form of the kernel Λ(r) and its Fourier transformation $\Lambda_{q}$. The kernel Λ captures dissipative interactions in the system and encodes both self-dissipation and cross-coupling effects. The first diagonal entry of Λ corresponds to the dissipation associated with mechanical motion (i.e. the *h*-variable), while the second diagonal entry accounts for the dissipation of electrical currents (i.e. the $P_{z}$-variable). The off-diagonal terms of Λ represent the coupling between mechanical motion and electrical activity, a feature that may play a critical role in certain cellular or active matter processes where electromechanical interactions are prominent.

The Fourier transform $\Lambda_{q}$ introduces wavevector dependence, reflecting the nonlocal nature of dissipation in the spatial domain. Physically, this q-dependence arises from hydrodynamic interactions and long-range electromechanical couplings, which are inherently nonlocal. Determining the precise form of $\Lambda_{q}$ generally requires solving a boundary value problem derived from the governing equations of the specific dissipation model under consideration—such as Stokes flow for viscous media or Maxwell’s equations for electrodynamic effects. For the purposes of this work, and to avoid unnecessary technical complexity, we adopt a simplified yet representative form of $\Lambda_{q}$, motivated by prior studies (cf. (42)). Specifically, we assume that


(9) $$\Lambda_{q}=\left[\begin{array}{cc} \frac{1}{4\pi \eta_{0}q} & 0\\ 0 & \frac{1}{R} \end{array}\right],$$

where $\eta_{0}$ denotes the viscosity of the ambient medium and *R* is an effective resistance associated with the electrical dissipation channel in the cell membrane. This choice reflects a physically reasonable approximation in which hydrodynamic dissipation exhibits long-range behavior (∼1/q), while electrical dissipation is modeled as local and isotropic.

#### Fluctuation spectrum with thermal noise

In the presence of just the thermal noise, the time evolution equation in 7 becomes


(10) $$\frac{\partial v_{q}(t)}{\partial t}=\Theta_{q}v_{q}(t)+F_{q}^{\mathrm{th}}(t).$$

We assume that the thermal noise is not correlated in time by choosing the correlation function matrix for the thermal noise force of the form:


(11) $$\langle F_{q}^{\mathrm{th}}(t)⊗F_{q^{\prime }}^{\mathrm{th}}(t^{\prime })\rangle =2B_{q}^{\mathrm{th}}\delta_{qq^{\prime }}\delta (t-t^{\prime }),$$

where $B_{q}^{\mathrm{th}}$ is a symmetric coefficient matrix encoding the strength of thermal noise. One can fix the form of $B_{q}^{\mathrm{th}}$ using the Fluctuation–Dissipation theorem (for each mode q) for the multivariable system given as (39):


(12) $$B_{q}^{\mathrm{th}}=-\frac{1}{2}\left(\Theta_{q}M_{q}^{\mathrm{th}}+M_{q}^{\mathrm{th}}\Theta_{q}^{⊺}\right).$$

Here, $M_{q}^{\mathrm{th}}$ is the fluctuation spectrum matrix in thermal equilibrium and in the absence of active noise, and is given as:


(13) $$\begin{array}{rl} M_{q}^{\mathrm{th}} & \;:\;=\langle v_{q}(t)⊗v_{q}(t)\rangle_{\mathrm{eq}}^{\mathrm{th}}\\ & \;=\left[\begin{array}{cc} \langle |h_{q}{|}^{2}\rangle_{\mathrm{eq}}^{\mathrm{th}} & \langle h_{q}(P_{z}{)}_{q}\rangle_{\mathrm{eq}}^{\mathrm{th}}\\ \langle h_{q}(P_{z}{)}_{q}\rangle_{\mathrm{eq}}^{\mathrm{th}} & \langle |(P_{z}{)}_{q}{|}^{2}\rangle_{\mathrm{eq}}^{\mathrm{th}} \end{array}\right], \end{array}$$

and (cf. (34))


(14) $$\langle |h_{q}{|}^{2}\rangle_{\mathrm{eq}}^{\mathrm{th}}=\frac{k_{B}T}{L^{2}q^{2}\left(\lambda +(\kappa_{b}-f^{2}/a_{z})q^{2}\right)},$$

(15) $$\langle h_{q}(P_{z}{)}_{q}\rangle_{\mathrm{eq}}^{\mathrm{th}}=\frac{k_{B}Tfq^{2}}{L^{2}a_{z}\left(\lambda +(\kappa_{b}-f^{2}/a_{z})q^{2}\right)},$$

(16) $$\langle |(P_{z}{)}_{q}{|}^{2}\rangle_{\mathrm{eq}}^{\mathrm{th}}=\frac{k_{B}T\left(\lambda +\kappa_{b}q^{2}\right)}{L^{2}a_{z}\left(\lambda +(\kappa_{b}-f^{2}/a_{z})q^{2}\right)}.$$

Here, $\langle (\cdot )\rangle_{\mathrm{eq}}^{\mathrm{th}}$ denotes the equilibrium statistical average (fluctuation spectrum) of fluctuating quantity (⋅) when only thermal fluctuations are present. The steps to derive 12 are as follows. We first obtain the solution for $v_{q}(t)$ that satisfies 10 by neglecting the effect of initial value term $v_{q}(0)$ that decays to zero over a long time, as (39):


(17) $$v_{q}(t)=\int_{0}^{t}ds\left(e^{(t-s)\Theta_{q}}\right)F_{q}^{\mathrm{th}}(s).$$

Using 17 and 11, we obtain fluctuation spectrum as:


(18) $$\langle v_{q}(t)⊗v_{q}(t)\rangle^{\mathrm{th}}=2\int_{0}^{t}ds\;e^{(t-s)\Theta_{q}}B_{q}^{\mathrm{th}}e^{(t-s)\Theta_{q}^{⊺}}.$$

The fluctuation spectrum in thermal equilibrium is identified as the fluctuation at t→+∞, and obtained as,


(19) $$M_{q}^{\mathrm{th}}=\langle v_{q}(t)⊗v_{q}(t)\rangle_{\mathrm{eq}}^{\mathrm{th}}=2\int_{0}^{+\infty }dt\;e^{t\Theta_{q}}B_{q}^{\mathrm{th}}e^{t\Theta_{q}^{⊺}}.$$

Using 19, we construct


(20) $$\begin{array}{rl} & \Theta_{q}M_{q}^{\mathrm{th}}+M_{q}^{\mathrm{th}}\Theta_{q}^{⊺}\\ & \;=2\int_{0}^{+\infty }dt\;\Theta_{q}e^{t\Theta_{q}}B_{q}^{\mathrm{th}}e^{t\Theta_{q}^{⊺}}+2\int_{0}^{+\infty }dt\;e^{t\Theta_{q}}B_{q}^{\mathrm{th}}e^{t\Theta_{q}^{⊺}}\Theta_{q}^{⊺}\\ & \;=2\int_{0}^{+\infty }dt\;\frac{d}{dt}[e^{t\Theta_{q}}B_{q}^{\mathrm{th}}e^{t\Theta_{q}^{⊺}}]\\ & \;={\left(2e^{t\Theta_{q}}B_{q}^{\mathrm{th}}e^{t\Theta_{q}^{⊺}}\right)}_{t\to +\infty }-2B_{q}^{\mathrm{th}}. \end{array}$$

Noticing that $\Theta_{q}$ is negative-definite, the upper limit at infinite time vanishes, and we recover the Fluctuation–Dissipation theorem in 12.

#### Fluctuation spectrum with thermal and active noise

To account for the effects of active noises, we assume that the active noise corresponding to polarization $P_{z}$ is zero and write the time-correlation function for the active noise $\xi_{q}^{a}$ in 8 as (cf. (42))


(21) $$\langle \xi_{q}^{a}(t)⊗\xi_{q^{\prime }}^{a}(t^{\prime })\rangle =\left[\begin{array}{cc} \Gamma_{q}^{a} & 0\\ 0 & 0 \end{array}\right]\delta_{qq^{\prime }}e^{-|t-t^{\prime }|/\tau^{a}}.$$

Here, $\Gamma_{q}^{a}$ is the strength of autocorrelation of active noise, and $\tau^{a}>0$ is a characteristic time-scale for exponentially time-decaying active noise. Using 8 and 21, we construct the correlation function matrix for the active noise as:


(22) $$\langle F_{q}^{a}(t)⊗F_{q^{\prime }}^{a}(t^{\prime })\rangle =\;:\;2B_{q}^{a}\;\delta_{qq^{\prime }}e^{-|t-t^{\prime }|/\tau^{a}},$$

where the symmetric matrix $B_{q}^{a}$ is given as:


(23) $$B_{q}^{a}=\left[\begin{array}{cc} (B_{q}^{a}{)}_{11} & 0\\ 0 & 0 \end{array}\right],\;(B_{q}^{a}{)}_{11}=\frac{1}{2}{\left(\frac{1}{4\pi \eta_{0}q}\right)}^{2}\Gamma_{q}^{a}.$$

Neglecting the effect of initial value term $v_{q}(0)$ that decays to zero over a long time, we obtain the solution for $v_{q}(t)$ that satisfies 7 as (39):


(24) $$v_{q}(t)=\int_{0}^{t}ds\left(e^{(t-s)\Theta_{q}}\right)\left(F_{q}^{\mathrm{th}}(s)+F_{q}^{a}(s)\right).$$

Using 24, and assuming thermal and active noises are uncorrelated with each other in time, we obtain the fluctuation spectrum matrix $\langle v_{q}(t)⊗v_{q}(t)\rangle^{\mathrm{th},a}$ when both thermal and active noise are present as:


(25) $$\begin{array}{rl} & \langle v_{q}(t)⊗v_{q}(t)\rangle^{\mathrm{th},a}=\int_{0}^{t}ds\int_{0}^{t}ds^{\prime }(e^{(t-s)\Theta_{q}})\\ & \;(F_{q}^{\mathrm{th}}(s)⊗F_{q}^{\mathrm{th}}(s^{\prime })+F_{q}^{a}(s)⊗F_{q}^{a}(s^{\prime }))(e^{(t-s^{\prime })\Theta_{q}^{⊺}})\\ & \;=\;:\;\langle v_{q}(t)⊗v_{q}(t)\rangle^{\mathrm{th}}+\langle v_{q}(t)⊗v_{q}(t)\rangle^{a}, \end{array}$$

where the thermal contribution $\langle v_{q}(t)⊗v_{q}(t)\rangle^{\mathrm{th}}$ is given by 18. In the presence of active noise, the system is out of equilibrium. For any fluctuating quantity (⋅), we obtain its steady state value in the presence of active noise by taking limit t→∞ and denote the steady state value by $(\cdot {)}_{\mathrm{ss}}$. Meanwhile, the contribution due to active noise at steady state is calculated as:


(26) $$\begin{array}{rl} & \langle v_{q}⊗v_{q}\rangle_{\mathrm{ss}}^{a}\\ & \;=\lim_{t\to +\infty }[\int_{0}^{t}ds\int_{0}^{t}ds^{\prime }(e^{(t-s)\Theta_{q}})(2B_{q}^{a}e^{-|s-s^{\prime }|/\tau^{a}})\\ & \;(e^{(t-s^{\prime })\Theta_{q}^{⊺}})],\\ & \;=\lim_{t\to +\infty }[\int_{0}^{t}ds\int_{0}^{s}ds^{\prime }(e^{(t-s)\Theta_{q}})(2B_{q}^{a}e^{-(s-s^{\prime })/\tau^{a}})\\ & \;(e^{(t-s^{\prime })\Theta_{q}^{⊺}})\\ & \;+\int_{0}^{t}ds\int_{s}^{t}ds^{\prime }(e^{(t-s)\Theta_{q}})(2B_{q}^{a}e^{-(s^{\prime }-s)/\tau^{a}})(e^{(t-s^{\prime })\Theta_{q}^{⊺}})], \end{array}$$

We analytically evaluate the right-hand side in 26 using Mathematica software by first evaluating the double integrations, and then obtaining the long-time limit t→+∞.

### Renormalization of membrane properties due to thermal noise

Here, we present the general formula for the effective material constants of the cell membrane affected by fluctuations (thermal or active) and explicitly present the expressions for the effective material constants when only thermal noise is present, as derived in (34). We consider the polarization ($P_{\mathrm{total}}$) and the out-of-plane displacement $\left(h_{\mathrm{total}}\right)$ of the cell membrane as the state variables and decompose them as, $\left(P_{\mathrm{total}},h_{\mathrm{total}})=(\bar{P},\bar{h})+(P,h\right)$, where $\left(\bar{P},\bar{h}\right)$ represent the average values and (P,h) represent the fluctuating part. By averaging over the fluctuating part (P,h) using statistical mechanics, it is shown that (see (34) for detailed calculations) the free energy $F[\bar{P},\bar{h}]$ of a cell membrane depends locally on $\left(\bar{P},\bar{h}\right)$ as (up to quadratic terms in $\left(\bar{P},\bar{h}\right)$) (34)^c^:


(27) $$\begin{array}{rl} & F[\bar{P},\bar{h}]=L^{2}\lambda^{\mathrm{eff}}+\int_{S}[\frac{a^{\mathrm{eff}}}{2}\left({\bar{P_{x}}}^{2}+{\bar{P_{y}}}^{2}\right)+\frac{a_{z}^{\mathrm{eff}}}{2}{\bar{P_{z}}}^{2}\\ & \;-E_{z}^{0}\bar{P_{z}}+f^{\mathrm{eff}}\bar{P_{z}}\Delta \bar{h}+\frac{\kappa_{b}^{\mathrm{eff}}}{2}(\Delta \bar{h}{)}^{2}\\ & \;+\kappa_{g}^{\mathrm{eff}}\text{det}(\nabla \nabla \bar{h})+\frac{\lambda^{\mathrm{eff}}}{2}|\nabla \bar{h}{|}^{2}], \end{array}$$

where $a^{\mathrm{eff}},a_{z}^{\mathrm{eff}},f^{\mathrm{eff}},\kappa_{b}^{\mathrm{eff}},\kappa_{g}^{\mathrm{eff}},\lambda^{\mathrm{eff}}$ are the renormalized and effective macroscopic material constants with fluctuations. The expressions for effective material constants under only thermal fluctuations are analytically derived using the harmonic Hamiltonian in 4 and the Gaussian integral formula as (see Ref. (34) for detailed calculations):


(28) $$\begin{array}{rl} (a^{\mathrm{eff}}{)}^{\mathrm{th}} & =a\left(1+\frac{1}{2}\langle |\nabla h{|}^{2}\rangle_{\mathrm{eq}}^{\mathrm{th}}\right)\\ & \approx a\left(1+\frac{k_{B}T}{4\pi (\kappa_{b}-f^{2}/a_{z})}\ln\;\frac{L}{t_{m}}\right),\\ (a_{z}^{\mathrm{eff}}{)}^{\mathrm{th}} & =a_{z}\left(1+\frac{1}{2}\langle |\nabla h{|}^{2}\rangle_{\mathrm{eq}}^{\mathrm{th}}\right)\\ & \approx a_{z}\left(1+\frac{k_{B}T}{4\pi (\kappa_{b}-f^{2}/a_{z})}\ln\;\frac{L}{t_{m}}\right),\\ (f^{\mathrm{eff}}{)}^{\mathrm{th}} & =f\left(1-\langle |\nabla h{|}^{2}\rangle_{\mathrm{eq}}^{\mathrm{th}}\right)\\ & \approx f\left(1-\frac{k_{B}T}{2\pi (\kappa_{b}-f^{2}/a_{z})}\ln\;\frac{L}{t_{m}}\right),\\ (\kappa_{b}^{\mathrm{eff}}{)}^{\mathrm{th}} & =\kappa_{b}\left(1-\frac{3}{2}\langle |\nabla h{|}^{2}\rangle_{\mathrm{eq}}^{\mathrm{th}}\right)\\ & \approx \kappa_{b}\left(1-\frac{3k_{B}T}{4\pi (\kappa_{b}-f^{2}/a_{z})}\ln\;\frac{L}{t_{m}}\right),\\ (\kappa_{g}^{\mathrm{eff}}{)}^{\mathrm{th}} & =\kappa_{g}\left(1-\frac{3}{2}\langle |\nabla h{|}^{2}\rangle_{\mathrm{eq}}^{\mathrm{th}}\right)\\ & \approx \kappa_{g}\left(1-\frac{3k_{B}T}{4\pi (\kappa_{b}-f^{2}/a_{z})}\ln\;\frac{L}{t_{m}}\right),\\ (\lambda^{\mathrm{eff}}{)}^{\mathrm{th}} & =\lambda \left(1-\frac{3\eta \alpha }{16\pi }\right)+\kappa_{b}\eta \xi^{2}\left(\frac{2\theta -3}{4\pi }\right)\\ & +\;\frac{k_{B}T}{4\pi }\left(q_{\max}^{2}-q_{\min}^{2}+\int_{q_{\min}}^{q_{\max}}\frac{\left(\nu^{2}+\frac{q^{2}}{1-\theta }\right)qdq}{\nu^{2}+q^{2}}\right), \end{array}$$

where $\langle |\nabla h{|}^{2}\rangle_{\mathrm{eq}}^{\mathrm{th}}\approx \frac{\eta \alpha }{2\pi }$ (34), and the constants θ,η,ν,α,ξ are defined as:


(29) $$\begin{array}{cc} & \theta =\;\frac{\;f^{2}}{a\kappa_{b}},\;\eta =\frac{k_{B}T}{\kappa_{b}(1-\theta )},\;\nu^{2}=\frac{\lambda }{\kappa_{b}(1-\theta )},\\ & \alpha =\int_{q_{\min}}^{q_{\max}}\frac{qdq}{\nu^{2}+q^{2}}=\frac{1}{2}\log\;\frac{\nu^{2}+q_{\max}^{2}}{\nu^{2}+q_{\min}^{2}},\\ & \xi^{2}=\int_{q_{\min}}^{q_{\max}}\frac{q^{3}dq}{\nu^{2}+q^{2}}=\frac{q_{\max}^{2}-q_{\min}^{2}}{2}-\frac{\nu^{2}}{2}\log\;\frac{\nu^{2}+q_{\max}^{2}}{\nu^{2}+q_{\min}^{2}}. \end{array}$$

Here, $q_{\min}$ and $q_{\max}$ are physically motivated cut-offs for the wave vector q such that $q\in [q_{\min},q_{\max}]$. Note that, θ,η,α are dimensionless and ν,ξ has dimension of 1/length. To obtain effective material constants with both thermal and active noise, one would replace $\langle |\nabla h{|}^{2}\rangle_{\mathrm{eq}}^{\mathrm{th}}$ in 28 with quantity $\langle |\nabla h{|}^{2}\rangle_{\mathrm{ss}}^{\mathrm{th},a}$ that denotes the fluctuation spectrum at steady state when both thermal and active noise are present.

### Energy harvesting due to active noise

In this section, we derive the energy harvested by the cell membrane due to active noise. Using 25, 26, and the definition $\langle v_{q}⊗v_{q}\rangle_{\mathrm{ss}}^{\mathrm{th},a}\;:\;=\left[\begin{array}{cc} \langle |h_{q}{|}^{2}\rangle_{\mathrm{ss}}^{\mathrm{th},a} & \langle h_{q}{\left(P_{z}\right)}_{q}\rangle_{\mathrm{ss}}^{\mathrm{th},a}\\ \langle h_{q}{\left(P_{z}\right)}_{q}\rangle_{\mathrm{ss}}^{\mathrm{th},a} & \langle |{\left(P_{z}\right)}_{q}{|}^{2}\rangle_{\mathrm{ss}}^{\mathrm{th},a} \end{array}\right]$, we obtain $\langle |h_{q}{|}^{2}\rangle_{\mathrm{ss}}^{\mathrm{th},a}$, the fluctuation spectrum at steady state for displacement *h* with both thermal and active noise as,


(30) $$\begin{array}{cc} & \langle |h_{q}{|}^{2}\rangle_{\mathrm{ss}}^{\mathrm{th},a}=\langle |h_{q}{|}^{2}\rangle_{\mathrm{eq}}^{\mathrm{th}}+T_{0},\\ & T_{0}\;:\;=2(B_{q}^{a}{)}_{11}\\ & \;\times \frac{\left[\left(\omega_{0}-\Theta_{11}\right)+\tau_{a}\Theta_{22}\left(\mathrm{Tr}(\Theta_{q})-\frac{1}{\tau_{a}}\right)\right]}{\left[\left(\omega_{0}-\Theta_{11}\right)\mathrm{Tr}(\Theta_{q})\left(\mathrm{Tr}(\Theta_{q})-\frac{1}{\tau_{a}}+\tau_{a}\Theta_{22}\left(\omega_{0}-\Theta_{11}\right)\right)\right]}. \end{array}$$

Here, we define $\omega_{0}\;:\;=\frac{\Theta_{12}\Theta_{21}}{\Theta_{22}}$, and $\Theta_{ij}$ are entries in the matrix $\Theta_{q}$ defined in 8. Quantity $\langle |h_{q}{|}^{2}\rangle_{\mathrm{eq}}^{\mathrm{th}}$ is the fluctuation spectrum for displacement *h* with only thermal noise, given in 14. We evaluate the analytical expression for $T_{0}$ in 30 using 26 under the following constraints:


(31) $$\begin{array}{rl} & \omega^{\prime }<\frac{2}{\tau_{a}},\\ & \;-\mathrm{Tr}(\Theta_{q})-\frac{\omega^{\prime }}{2}>\frac{1}{\tau_{a}},\\ & \;3\tau_{a}(\omega^{\prime }{)}^{2}-8\tau_{a}\mathrm{Tr}(\Theta_{q})\left(\frac{1}{2\tau_{a}}-\mathrm{Tr}(\Theta_{q})\right)\\ & \;-10\tau_{a}\omega^{\prime }\left(\frac{2}{5\tau_{a}}-\mathrm{Tr}(\Theta_{q})\right)>\frac{4}{\tau_{a}},\\ & \;-3\tau_{a}(\omega^{\prime }{)}^{2}+4\tau_{a}\mathrm{Tr}(\Theta_{q})\left(\frac{2}{\tau_{a}}-\mathrm{Tr}(\Theta_{q})\right)\\ & \;+8\tau_{a}\omega^{\prime }\left(\frac{1}{\tau_{a}}-\mathrm{Tr}(\Theta_{q})\right)<\frac{4}{\tau_{a}}, \end{array}$$

where we define $\omega^{\prime }\;:\;=\sqrt{4\Theta_{12}\Theta_{21}+(\Theta_{11}-\Theta_{22}{)}^{2}}$. We note that the quantities $\Theta_{11},\Theta_{22},\mathrm{Tr}(\Theta_{q}),\omega_{0},\omega^{\prime }$ have unit of frequency (i.e. 1/sec) and correspond to cell membrane time-dynamics. Hence, the constraints in 31 signify the conditions that these frequencies relating to cell membrane time dynamics shall satisfy with respect to frequency for active noise forcing $\left(1/\tau_{a}\right)$ for stable steady state properties of the cell membrane. We ensure that the above constraints are satisfied by the model parameter values we choose (Table 1) for a typical cell membrane and for a chosen range of $q\in [q_{\min},q_{\max}]$. We define the term $S_{0}$ as the contribution of the active noise in the statistical average term $\langle |\nabla h{|}^{2}\rangle_{\mathrm{eq}}$, obtained as (28, 34):


(32) $$\begin{array}{rl} S_{0} & \;:\;=\langle |\nabla h{|}^{2}\rangle_{\mathrm{ss}}^{\mathrm{th},a}-\langle |\nabla h{|}^{2}\rangle_{\mathrm{eq}}^{\mathrm{th}},\\ & =\sum_{q\in K}q^{2}\left(\langle |h_{q}{|}^{2}\rangle_{\mathrm{ss}}^{\mathrm{th},a}-\langle |h_{q}{|}^{2}\rangle_{\mathrm{eq}}^{\mathrm{th}}\right),\\ & =\sum_{q\in K}q^{2}T_{0}\;(\text{using 30}),\\ & =\left(\frac{L^{2}}{2\pi }\right)\int_{q_{\min}}^{q_{\max}}dq\;q^{3}T_{0}. \end{array}$$

The cut-off maximum wave vector $q_{\max}$ is related to the membrane thickness $t_{m}$ and chosen as $q_{\max}=\frac{2\pi }{t_{m}}$, and $q_{\min}$ is dictated by the macroscopic coarse-graining length scale *L* as $q_{\min}=\frac{2\pi }{L}$ (34). Using $S_{0}$ in 32 and 28, we finally obtain the expressions for the effective macroscopic material properties for cell membrane, $(a^{\mathrm{eff}}{)}^{\mathrm{th},a},(a_{z}^{\mathrm{eff}}{)}^{\mathrm{th},a},(f^{\mathrm{eff}}{)}^{\mathrm{th},a},(\kappa_{b}^{\mathrm{eff}}{)}^{\mathrm{th},a},(\kappa_{g}^{\mathrm{eff}}{)}^{\mathrm{th},a}$, and $(\lambda^{\mathrm{eff}}{)}^{\mathrm{th},a}$ with both thermal and active noise as:


(33) $$\begin{array}{cc} & (a^{\mathrm{eff}}{)}^{\mathrm{th},a}=(a^{\mathrm{eff}}{)}^{\mathrm{th}}+aS_{0}/2,\\ & (a_{z}^{\mathrm{eff}}{)}^{\mathrm{th},a}=(a_{z}^{\mathrm{eff}}{)}^{\mathrm{th}}+a_{z}S_{0}/2,\\ & (f^{\mathrm{eff}}{)}^{\mathrm{th},a}=(f^{\mathrm{eff}}{)}^{\mathrm{th}}-fS_{0},\\ & (\kappa_{b}^{\mathrm{eff}}{)}^{\mathrm{th},a}=(\kappa_{b}^{\mathrm{eff}}{)}^{\mathrm{th}}-3\kappa_{b}S_{0}/2,\\ & (\kappa_{g}^{\mathrm{eff}}{)}^{\mathrm{th},a}=(\kappa_{g}^{\mathrm{eff}}{)}^{\mathrm{th}}-3\kappa_{g}S_{0}/2,\\ & (\lambda^{\mathrm{eff}}{)}^{\mathrm{th},a}=(\lambda^{\mathrm{eff}}{)}^{\mathrm{th}}-3\lambda S_{0}/8-3\kappa_{b}S_{0}/2. \end{array}$$

Here, the expressions for $(a^{\mathrm{eff}}{)}^{\mathrm{th}},(a_{z}^{\mathrm{eff}}{)}^{\mathrm{th}},(f^{\mathrm{eff}}{)}^{\mathrm{th}},(\kappa_{b}^{\mathrm{eff}}{)}^{\mathrm{th}},(\kappa_{g}^{\mathrm{eff}}{)}^{\mathrm{th}}$, and $(\lambda^{\mathrm{eff}}{)}^{\mathrm{th}}$, the effective material properties with only thermal noise are given in 28. Using 33, and the free energy *F* in 27, we obtain $\Delta F^{a}$, the contribution of active noise in the effective free energy $F[\bar{P},\bar{h}]$ at steady state as:


(34) $$\begin{array}{rl} & \Delta F^{a}=F[\bar{P},\bar{h}{]}^{\mathrm{th},a}-F[\bar{P},\bar{h}{]}^{\mathrm{th}}\\ & \;=\int_{S}[\frac{aS_{0}}{4}\left((\bar{P_{x}}{)}_{\mathrm{ss}}^{2}+(\bar{P_{y}}{)}_{\mathrm{ss}}^{2}\right)+\frac{a_{z}S_{0}}{4}(\bar{P_{z}}{)}_{\mathrm{ss}}^{2}\\ & \;-fS_{0}(\bar{P_{z}}{)}_{\mathrm{ss}}(\Delta \bar{h}{)}_{\mathrm{ss}}-(3\kappa_{b}S_{0}/4)(\Delta \bar{h}{)}_{\mathrm{ss}}^{2}\\ & \;-(3\kappa_{g}S_{0}/2)\text{det}(\nabla \nabla \bar{h}{)}_{\mathrm{ss}}\\ & \;-\frac{1}{2}\left(3\lambda S_{0}/8+3\kappa_{b}S_{0}/2\right)|\nabla \bar{h}{|}_{\mathrm{ss}}^{2}]\\ & \;-L^{2}\left(3\lambda S_{0}/8+3\kappa_{b}S_{0}/2\right). \end{array}$$

Here, as stated earlier, $\bar{h}$ and $\bar{P}=[\bar{P_{x}},\bar{P_{y}},\bar{P_{z}}]$, respectively, are nonfluctuating average values of out-of-plane displacement and polarization of the cell membrane. $\Delta F^{a}$ (assuming $\Delta F^{a}>0$) is the energy harvested by the cell membrane from the active noise at steady state.

**Table 1.** Numerical values of model parameters used for plot in Fig. 4.

Parameter	Value
Thickness of cell membrane $\left(t_{m}\right)$	5 nm (75)
Length of cell membrane (L)	10 μm (75)
Bending modulus of cell membrane $\left(\kappa_{b}\right)$	15 $k_{B}T$ (34)
Surface tension of cell membrane (λ)	$50\times 10^{-3}$ N/m (76)
Dielectric permittivity of cell membrane (ϵ)	$20\epsilon_{0}$ F/m (75, 77)
Flexoelectric coupling constant of cell membrane $\left(f_{e}\right)$	$0.32\times 10^{-19}$ C (34, 78)
Effective electrical resistance of cell membrane (R)	145MΩ (79)
Resting transmembrane voltage $\left(({\bar{V}}_{z}{)}_{\mathrm{eq}}^{\mathrm{th}}\right)$	−60 mV (80)
Characteristic time-scale for active protein force noise $\left(\tau_{a}\right)$	0.15 ns (81)
Number of proteins exerting active force $\left(N_{p}\right)$	500 (72, 82)
Viscosity of embedding fluid $\left(\eta_{0}\right)$	$2\times 10^{-3}\;\mathrm{Pa}\cdot s$ (83)
Boltzmann constant $\left(k_{B}\right)$	$1.38\times 10^{-23}\;J/K$
Vacuum permittivity $\left(\epsilon_{0}\right)$	$8.854\times 10^{-12}\;F/m$
Temperature (T)	298K

### Changes in transmembrane voltage due to active noise

We re-write the free energy in 27 up to quadratic terms of $\left(\bar{P_{z}},\Delta \bar{h}\right)$ as (34),


(35) $$\begin{array}{rl} F[\bar{P},\bar{h}] & =L^{2}\lambda^{\mathrm{eff}}+\int_{S}[\frac{a^{\mathrm{eff}}}{2}\left({\bar{P_{x}}}^{2}+{\bar{P_{y}}}^{2}\right)+\frac{a_{z}^{\mathrm{eff}}}{2}{\bar{P_{z}}}^{2}\\ & \;-E_{z}^{0}\bar{P_{z}}+f^{\mathrm{eff}}\bar{P_{z}}\Delta \bar{h}+\frac{\kappa_{b}^{\mathrm{eff}}}{2}(\Delta \bar{h}{)}^{2}]. \end{array}$$

Then, we minimize the free energy in 35 with respect to variables $\left(\bar{P_{z}},\Delta \bar{h}\right)$ and obtain the expressions for energy minimizing steady state polarization $(\bar{P_{z}}{)}_{\mathrm{ss}}$ and steady state curvature $(\Delta \bar{h}{)}_{\mathrm{ss}}$ as,


(36) $$\begin{array}{cc} & ({\bar{P}}_{z}{)}_{\mathrm{ss}}=\frac{E_{z}^{0}\kappa_{b}^{\mathrm{eff}}}{a_{z}^{\mathrm{eff}}\kappa_{b}^{\mathrm{eff}}-(f^{\mathrm{eff}}{)}^{2}},\\ & (\Delta \bar{h}{)}_{\mathrm{ss}}=-\frac{E_{z}^{0}f^{\mathrm{eff}}}{a_{z}^{\mathrm{eff}}\kappa_{b}^{\mathrm{eff}}-(f^{\mathrm{eff}}{)}^{2}}. \end{array}$$

The change in equilibrium polarization $({\bar{P}}_{z}{)}_{\mathrm{eq}}$ at steady state due to active noise is obtained as:


(37) $$\begin{array}{rl} ({\bar{P}}_{z}{)}_{\mathrm{ss}}^{\mathrm{th},a}-({\bar{P}}_{z}{)}_{\mathrm{eq}}^{\mathrm{th}} & =\frac{E_{z}^{0}(\kappa_{b}^{\mathrm{eff}}{)}^{\mathrm{th},a}}{(a_{z}^{\mathrm{eff}}{)}^{\mathrm{th},a}(\kappa_{b}^{\mathrm{eff}}{)}^{\mathrm{th},a}-((f^{\mathrm{eff}}{)}^{\mathrm{th},a}{)}^{2}}\\ & \;-\frac{E_{z}^{0}(\kappa_{b}^{\mathrm{eff}}{)}^{\mathrm{th}}}{(a_{z}^{\mathrm{eff}}{)}^{\mathrm{th}}(\kappa_{b}^{\mathrm{eff}}{)}^{\mathrm{th}}-((f^{\mathrm{eff}}{)}^{\mathrm{th}}{)}^{2}}, \end{array}$$

where the expressions for effective material properties with only thermal noise are given in 28 and effective material properties with both thermal and active noise are given in 33. The finite change in equilibrium polarization in 37 due to active noise at steady state indicates that the charges on the cell membrane walls facing intracellular and extracellular fluids have changed due to active biological processes.

The transmembrane voltage is dictated by the combined activity of ion concentration gradient in extracellular and intracellular fluids, numerous ion channels, pumps, and gap junction complexes (70). We use a sign convention for ${\bar{V}}_{z}$, the transmembrane voltage of cell membrane as (71):


(38) $${\bar{V}}_{z}=\phi_{i}-\phi_{e},$$

where $\phi_{i}$ and $\phi_{e}$ are electric potentials of cell membrane walls facing intracellular and extracellular fluids, respectively. We choose the direction pointing from intracellular fluid to extracellular fluid as the +ve *z* direction. We introduce a notation $({\bar{P}}_{z}{)}_{\mathrm{ss}}\;:\;=({\bar{P}}_{z}{)}_{\mathrm{ss}}\hat{z}$ as the steady state polarization in the cell membrane in *z* direction and $\hat{z}$ is a unit orientation vector in +ve *z* direction. Assuming the cell membrane as a linear dielectric material, a linear relationship between the electric field in the cell membrane $E_{z}^{0}=E_{z}^{0}\hat{z}$ and the induced steady state polarization in the membrane $({\bar{P}}_{z}{)}_{\mathrm{ss}}$ is given by:


(39) $$\begin{array}{cc} & E_{z}^{0}\hat{z}=a_{z}\left(({\bar{P}}_{z}{)}_{\mathrm{ss}}\hat{z}\right),\\ & E_{z}^{0}\;:\;=-\left(\frac{\phi_{e}-\phi_{i}}{t_{m}}\right). \end{array}$$

Using 38 and 39, we convert the change in polarization due to active noise at steady state in 37 into the change in the transmembrane voltage due to active noise at steady state, $({\bar{V}}_{z}{)}_{\mathrm{ss}}^{\mathrm{th},a}-({\bar{V}}_{z}{)}_{\mathrm{eq}}^{\mathrm{th}}$, as:


(40) $$({\bar{V}}_{z}{)}_{\mathrm{ss}}^{\mathrm{th},a}-({\bar{V}}_{z}{)}_{\mathrm{eq}}^{\mathrm{th}}=(a_{z}t)\left(({\bar{P}}_{z}{)}_{\mathrm{ss}}^{\mathrm{th},a}-({\bar{P}}_{z}{)}_{\mathrm{eq}}^{\mathrm{th}}\right).$$

## Discussion

With the results from the preceding sections, we are now equipped to analyze the biophysical implications of active noise and the subsequent flexoelectricity-mediated energy transduction.

### Active ion transport using harvested energy

**Fig. 2.**
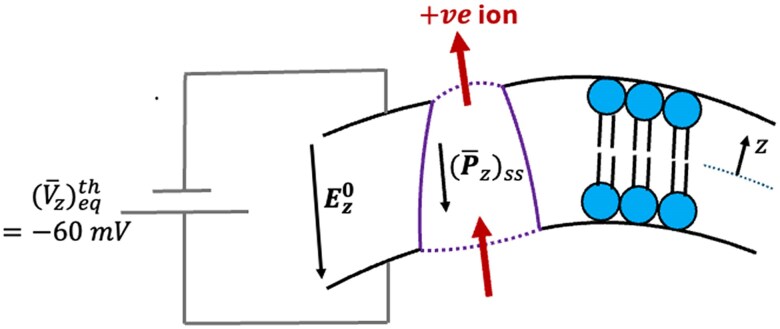
Active ion transport mechanism when cell membrane polarization $P_{z}$ increases due to active protein force noise. Due to an increase in steady state polarization $({\bar{P}}_{z}{)}_{\mathrm{ss}}$ in the cell membrane, the +ve ion shall transport from intracellular to extracellular fluid. Since the +ve ion is transported in the opposite direction of the transmembrane electric field $E_{z}^{0}$, this results in an *active* proton pumping mechanism.

Figure 2 highlights the central concept underpinning the active ion transport mechanism when the steady state polarization of cell membrane, $({\bar{P}}_{z}{)}_{\mathrm{ss}}$, increases due to active noise. To the best of our knowledge, the possibility of this mechanism (qualitatively) was first pointed out by Petrov (22). For a typical −ve resting transmembrane voltage, the corresponding electric field $E_{z}^{0}$ and induced polarization $({\bar{P}}_{z}{)}_{\mathrm{ss}}$ in a dielectric cell membrane, both point from extracellular to intracellular fluid region, as shown in Fig. 2. When the active noise increases the polarization $({\bar{P}}_{z}{)}_{\mathrm{ss}}$ in an ion-transporting protein globule of the cell membrane, a +ve ion must transport from the inner end (intracellular fluid side) to the outer end (extracellular fluid side) of the protein globule. This transported +ve ion must then exit from the outer wall of the cell membrane into the extracellular fluid. Effectively, an increase in $({\bar{P}}_{z}{)}_{\mathrm{ss}}$ due to active noise has resulted in a transport of +ve ions from intracellular to extracellular fluid in the opposite direction of transmembrane electric voltage $E_{z}^{0}$. Hence, this is an *active* proton pumping that the cell membrane performs using the energy harvested through active noise.

**Fig. 3.**
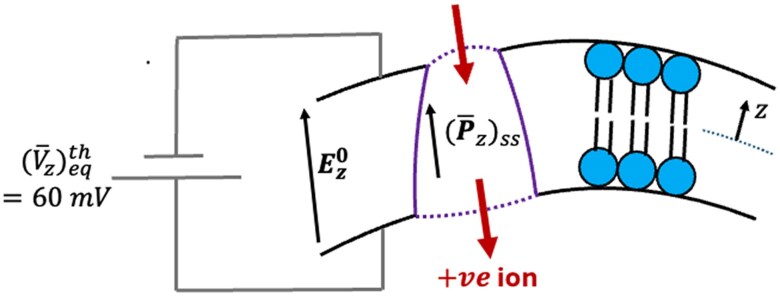
Active ion transport mechanism when membrane polarization $P_{z}$ decreases due to active protein force noise. Due to a decrease in steady state polarization $({\bar{P}}_{z}{)}_{\mathrm{ss}}$ in the cell membrane, the +ve ion shall transport from extracellular to intracellular fluid. Since the +ve ion is transported in the opposite direction of the transmembrane electric field $E_{z}^{0}$, this results in an *active* proton pumping mechanism.

Figure 3 highlights the active ion transport mechanism when the steady state cell membrane polarization $({\bar{P}}_{z}{)}_{\mathrm{ss}}$ decreases due to active noise. When the resting transmembrane voltage is +ve, the corresponding electric field $E_{z}^{0}$ and induced polarization $({\bar{P}}_{z}{)}_{\mathrm{ss}}$ in a dielectric cell membrane, both point from intracellular to extracellular fluid region, as shown in Fig. 3. When the active noise decreases the polarization $({\bar{P}}_{z}{)}_{\mathrm{ss}}$ in an ion-transporting protein globule of the cell membrane, a +ve ion must transport from the outer end (extracellular fluid side) to the inner end (intracellular fluid side) of the protein globule. This transported +ve ion must then exit from the inner wall of the cell membrane into the intracellular fluid. Effectively, a decrease in $({\bar{P}}_{z}{)}_{\mathrm{ss}}$ due to active noise has resulted in a transport of +ve ions from extracellular to intracellular fluid in the opposite direction of transmembrane electric voltage $E_{z}^{0}$. Hence, this is an *active* proton pumping that the cell membrane performs using the energy harvested through active noise.

We consider that the active noise originates from the spatially varying curvature-induced active force exerted by active proteins in a cell membrane and model the strength of autocorrelation of active noise $\Gamma_{q}^{a}$ defined in 21 as (42, 72):


(41) $$\begin{array}{cc} & \Gamma_{q}^{a}=\rho_{a}mq^{4};\\ & \rho_{a}=N_{p}/L^{2},\;m=(\kappa_{b}c_{0}b^{2}{)}^{2}, \end{array}$$

where $\rho_{a}$ is the area density of active proteins, $N_{p}$ is the number of proteins exerting active force, m>0 is the curvature-induced active force parameter, $c_{0}$ is positive or negative spontaneous curvature of the cell membrane, and $b^{2}$ is the area occupied by the active proteins.

**Fig. 4.**
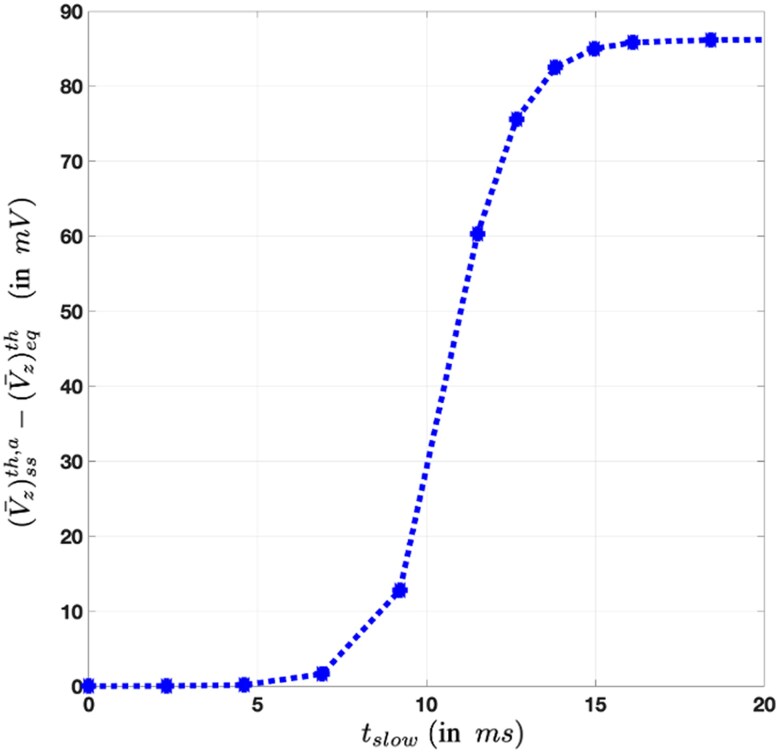
Changes in equilibrium transmembrane voltage of $({\bar{V}}_{z}{)}_{\mathrm{eq}}^{\mathrm{th}}=-60\;\mathrm{mV}$ due to active protein force noise at steady state. The *x*-axis is a timescale $\left(t_{\mathrm{slow}}\right)$ in millisecond, which is assumed to be proportional to $\log\;(m/m_{0})$. Here, $m=(\kappa_{b}c_{0}b^{2}{)}^{2}$ is the curvature-induced active protein force parameter (see 41), and constant $m_{0}$ is a reference value chosen for parameter *m* evaluated for temperature T=298K, bending modulus $\kappa_{b}=15k_{B}T$, spontaneous curvature $c_{0}=0.02\;{\mathrm{nm}}^{-1}$, area occupied by active proteins being 25% of total membrane area (i.e. $b^{2}=0.25L^{2}$). Changes in equilibrium transmembrane voltage at steady state are in the physiological range for a range of active biological processes, such as active ion transport. The nonlinear rise in −ve transmembrane voltage over millisecond timescale is consistent with the typical action potential curve for neurons (71, 84–86), which also serves as a foundational motivation behind nonlinear activation functions used in artificial neural networks (ANNs) (62–64, 87) for computing.

Table 1 presents the numerical values of various parameters used for plotting the numerical result. All the chosen numerical values lie in the physiological range for a living cell membrane. Using 37 and $S_{0}$ expression in 32, we evaluate the change in transmembrane voltage at steady state derived in 40. Integration in *q*-space in the $S_{0}$ expression in 32 is performed numerically using MATLAB. Figure 4 shows the changes in the typical −ve transmembrane voltage of −60mV as the curvature-induced active force activity exponentially increases with millisecond timescale. It is observed that the −ve transmembrane voltage can be increased by up to 90 mV at steady state due to active force activity. This wide range of changes in the transmembrane voltage due to active noise shows that active protein force is capable of performing a range of active biological functions, including active ion transport (65, 70, 73, 74).

We note that, both an elevated temperature and active noise increase the amplitude of membrane fluctuations and renormalize electromechanical properties. However, an elevated temperature alone cannot lead to a violation of detailed balance and thus cannot produce a net directional flux of ions. Active noise, on the other hand, breaks the detailed balance and hence injects extra energy into the cell membrane to enable active directional processes such as sustained ion pumping (58, 59).

### Action potential curve of neurons

Here, we highlight how the nonlinear variation in transmembrane voltage in millisecond timescale due to active protein force matches the typical nonlinear action potential curve of neuron cells during neuronal firing. The time dynamics arising from the active protein forces occur over various timescales during living cell functioning. For example, active protein dynamics, interactions, and chemical reactions occur over nanoseconds to microseconds timescale (81). Whereas mechanisms such as active ion transport and the voltage changes in cells like neurons during nerve impulses are typically observed over a relatively slower timescale $\left(t_{\mathrm{slow}}\right)$ in milliseconds (84–86). In this work, we have chosen the characteristic timescale $\tau_{a}$ of curvature-induced active protein force in nanoseconds. We take a long-time limit over this nanosecond timescale to obtain the steady state properties of a cell membrane, such as active noise induced changes in transmembrane voltage varying over a slow timescale $\left(t_{\mathrm{slow}}\right)$ chosen as milliseconds. It is experimentally evident that the electrical currents in neuron cells through BK calcium-activated potassium channels (Fig. 1C in Ref. (88)) and T-type calcium channels (Fig. 3 in Ref. (67)) rise exponentially over a millisecond timescale when the −ve transmembrane voltage increases nonlinearly in the action potential curve during neuronal firing. We assume that this electrical current response of ion channels linearly correlates with active protein force activity and model that the strength of active protein force noise also rises exponentially over a slow timescale $\left(t_{\mathrm{slow}}\right)$, i.e. $t_{\mathrm{slow}}\propto \log\;(m/m_{0})$ during neuronal firing. Here, $m_{0}$ is some reference value for the active protein force parameter *m*. The nonlinear variation in equilibrium transmembrane voltage in Fig. 4 over millisecond timescale obtained using our model matches quantitatively as well as qualitatively with the nonlinear rise in −ve transmembrane voltage over millisecond timescale in a typical action potential curve of neuron cells (62, 71, 84–86). The exponential-like rise of transmembrane voltage as observed in Fig. 4 during initiation of action potential resembles the voltage signature of an RC circuit, a familiar electrodynamic model for cell membranes (89–92), during capacitor charging. This is also consistent with our assumption that electrical dissipation of the dielectric cell membrane occurs through its effective electrical resistance (see Ref. 9). This nonlinear rise in −ve transmembrane voltage in an action potential curve of neurons is a fundamental motivation behind nonlinear *activation functions* (e.g. sigmoid, *tanh*, and rectified linear unit (ReLU)) used in artificial neural networks (63, 64, 87). Thus (speculatively), our thesis may provide a potential link between the two broad worlds of brain neuron functioning and artificial neural networks (62).

### Condition for polarity of transmembrane voltage and direction of ion transport

A key thing to note is that an increase in polarization $({\bar{P}}_{z}{)}_{\mathrm{ss}}$ needs the transmembrane voltage to be −ve (see sign convention in 38) for active noise to perform active proton pumping, and a decrease in polarization $({\bar{P}}_{z}{)}_{\mathrm{ss}}$ needs the transmembrane voltage to be +ve for active noise to perform active proton pumping. We use 37 that dictates the sign of change in $({\bar{P}}_{z}{)}_{\mathrm{ss}}$ to obtain the condition for the polarity of the transmembrane voltage. Doing simple algebra, we derive the condition (up to first order in $S_{0}$) to be satisfied for transmembrane voltage to be −ve as:


(42) $$S_{0}>0.$$

Note that $S_{0}$ in 32 is nondimensional and depends on the material parameters of the cell membrane and the nature of the active protein force (expression for $\Gamma_{q}^{a}$). As explained in the earlier subsection, when transmembrane voltage is −ve, active proton transport corresponds to the +ve ion being transported from the intracellular to the extracellular fluid, and when transmembrane voltage is +ve, active proton transport corresponds to the +ve ion being transported from the extracellular to the intracellular fluid. Hence, along with the sign of the transmembrane voltage, the condition in 42 also dictates the direction of active proton pumping across the cell membrane. The condition in 42 provides insight into how the polarity of transmembrane voltage and direction of ion transport is affected by the complex interplay between factors such as electric and elastic material constants of the cell membrane, ion concentration in extracellular and intracellular regions, changes in the molecular structure of a cell membrane (e.g. conformational changes in internal protein globules (22)), and active biological functioning such as ion transport.

We note that, although the fundamental flexoelectric coupling is linear in polarization and membrane curvature in the Hamiltonian (see 2), the effective steady state response is nonlinear (e.g. change in polarization of the membrane is nonlinear in the renormalized flexoelectric coupling constant $f^{\mathrm{eff}}$, as shown in 37) when the active noise is present. This emergent nonlinearity in the steady state response of the cell membrane is capable of producing directed effects such as directional flux of ions when detailed balance is broken in the presence of active noise, despite nondirectional nature of active fluctuations (58, 60, 93, 94).

## Concluding remarks

In this work, we developed a nonequilibrium statistical-mechanics framework to reveal how flexoelectricity-mediated time-correlated active protein forces transform the electromechanical behavior of cell membranes. We show that living membranes can harvest mechanical activity to generate physiologically significant transmembrane voltages and actively pump ions against their electrochemical gradient. Our model captures the hallmark nonlinear rise in negative membrane potential—mimicking neuronal action potentials—and even provides a biophysical underpinning for the activation functions at the heart of neural computation. We have proposed a simple criterion that relates membrane elastic and dielectric properties, together with active-force parameters, to the polarity and direction of ion transport.

Extending this framework to multicellular assemblies (95) with perhaps the inclusion of instabilities (96) could illuminate how active fluctuations drive collective bioelectric phenomena at the tissue scale. Investigating electromechanical dynamics in neuron networks may bridge molecular flexoelectricity and complex information processing, with implications for both understanding brain function and discovering bio-inspired computational materials.

## Data Availability

The data underlying this article are available in the article.

## Notes

a The bending modulus $\kappa_{b}$ for a cell membrane is in the range of $10k_{B}T$ to $20k_{B}T$, where $k_{B}$ is a Boltzmann constant and *T* is temperature in *K* (34, 97).

b Note that the term $\kappa_{g}\text{det}(\nabla \nabla h)$ does not contribute to the Hamiltonian H[P,h] in spatial Fourier (q) space due to the Gauss–Bonnet theorem (34), hence, we have neglected it.

c Note that, the resting transmembrane electric field $\left(E_{z}^{0}\right)$ is unchanged due to thermal or active fluctuations. This is because the statistical average of linear term $-E_{z}^{0}P_{z}$ in $P_{z}$ using harmonic Hamiltonian in 4 having up to quadratic terms in (P,h) is zero (see derivation in (34) for statistical average computation).
